# SARS-CoV-2 immunity and functional recovery of COVID-19 patients 1-year after infection

**DOI:** 10.1038/s41392-021-00777-z

**Published:** 2021-10-13

**Authors:** Yan Zhan, Yufang Zhu, Shanshan Wang, Shijun Jia, Yunling Gao, Yingying Lu, Caili Zhou, Ran Liang, Dingwen Sun, Xiaobo Wang, Zhibing Hou, Qiaoqiao Hu, Peng Du, Hao Yu, Chang Liu, Miao Cui, Gangling Tong, Zhihua Zheng, Yunsheng Xu, Linyu Zhu, Jin Cheng, Feng Wu, Yulan Zheng, Peijun Liu, Peng Hong

**Affiliations:** 1 Department of Rehabilitation Medicine, Xiangyang Central Hospital, Affiliated Hospital of Hubei University of Arts and Science, Xiangyang, Hubei 441021 China; 2 Department of Laboratory Medicine, Xiangyang Central Hospital, Affiliated Hospital of Hubei University of Arts and Science, Xiangyang, Hubei 441021 China; 3 Department of Radiology and Medical Imaging, Xiangyang Central Hospital, Affiliated Hospital of Hubei University of Arts and Science, Xiangyang, Hubei 441021 China; 4 Department of Research Affairs, Xiangyang Central Hospital, Affiliated Hospital of Hubei University of Arts and Science, Xiangyang, Hubei 441021 China; 5 Department of Nephrology, Center of Nephrology and Urology, Sun Yat-sen University Seventh Hospital, Shenzhen, Guangdong 518107 China; 6 Department of Biomedical Science, Shenzhen Research Institute, City University of Hong Kong, Kowloon Tong, Hong Kong, China; 7 Department of Rehabilitation Medicine, Xiangzhou District People’s Hospital, Xiangyang, Hubei 441000 China; 8 Department of Rehabilitation Medicine, Gucheng People’s Hospital, Affiliated Gucheng Hospital of Hubei University of Arts and Science, Xiangyang, Hubei 441700 China; 9 Division of Quality Control, Xiangyang Central Blood Station, Xiangyang, Hubei 441000 China; 10 Department of Respiratory and Critical Care Medicine, Xiangyang Central Hospital, Affiliated Hospital of Hubei University of Arts and Science, Xiangyang, Hubei 441021 China; 11 Department of Pathology, Mount Sinai St. Luke’s Roosevelt Hospital Center, New York, NY 10025 USA; 12 Department of Oncology, Peking University Shenzhen Hospital, Shenzhen, Guangdong 518036 China; 13 Department of Dermatology, Sun Yat-sen University Seventh Hospital, Shenzhen, Guangdong 518107 China; 14 Division of Research and Development, US Department of Veterans Affairs New York Harbor Healthcare System, Brooklyn, NY 11209 USA; 15 Department of Cell Biology, State University of New York Downstate Health Sciences University, Brooklyn, NY 11203 USA

**Keywords:** Infectious diseases, Adaptive immunity, Outcomes research

## Abstract

The long-term immunity and functional recovery after SARS-CoV-2 infection have implications in preventive measures and patient quality of life. Here we analyzed a prospective cohort of 121 recovered COVID-19 patients from Xiangyang, China at 1-year after diagnosis. Among them, chemiluminescence immunoassay-based screening showed 99% (95% CI, 98–100%) seroprevalence 10–12 months after infection, comparing to 0.8% (95% CI, 0.7–0.9%) in the general population. Total anti-receptor-binding domain (RBD) antibodies remained stable since discharge, while anti-RBD IgG and neutralization levels decreased over time. A predictive model estimates 17% (95% CI, 11–24%) and 87% (95% CI, 80–92%) participants were still 50% protected against detectable and severe re-infection of WT SARS-CoV-2, respectively, while neutralization levels against B.1.1.7 and B.1.351 variants were significantly reduced. All non-severe patients showed normal chest CT and 21% reported COVID-19-related symptoms. In contrast, 53% severe patients had abnormal chest CT, decreased pulmonary function or cardiac involvement and 79% were still symptomatic. Our findings suggest long-lasting immune protection after SARS-CoV-2 infection, while also highlight the risk of immune evasive variants and long-term consequences for COVID-19 survivors.

## Introduction

It has been more than one year since the first reported case of the novel coronavirus disease 2019 (COVID-19), which has already cost more than 2 million lives globally.^1^ Fortunately, vaccines against severe acute respiratory syndrome coronavirus 2 (SARS-CoV-2) have been developed with record-breaking speed and vaccine programs are ongoing worldwide to take the pandemic under control.^2^ During this expansion of research focus from treatment to prevention of COVID-19, the immune evasion mechanism and immunopathogenic nature of SARS-CoV-2 adds uncertainty to the efficacy of this global vaccination effort.^3^ During natural infection, SARS-CoV-2 could avoid the innate antiviral response mediated by interferons (IFNs) via an array of possible strategies,^4,5^ which not only leads to viral replication and spreading but also could delay or impair the adaptive immune response including T cell and antibody responses.^6,7^ The significant prevalence of SARS-CoV-2 RNA re-positive cases among discharged patients further raises the concern about the effectiveness and persistency of immune responses after natural infection.^8,9^ Recent long-term follow-up surveys report significant decrease of SARS-CoV-2 antibody titers 5 to 8 months after infection,^10–12^ but its correlation with reduced capacity of SARS-CoV-2 neutralization and immune memory is still debatable.^13,14^

Besides vaccination, equally important is the recovery and rehabilitation of COVID-19 patients.^15^ Mild cases usually do not require hospitalization but may share similar long-lasting symptoms or discomforts with severe cases, which may reduce life quality after recovery from COVID-19.^16,17^ Also, cardiac magnet resonance imaging (cMRI) screening revealed surprisingly high prevalence of subclinical myocardial inflammation and fibrosis in recently recovered patients.^18^ Due to the overloading of medical systems and the fear of in-hospital transmission, long-term follow-up studies of the structural and functional recovery of COVID-19-involved organs are still limited.^19^

In this prospective cohort study of recovered COVID-19 patients from Xiangyang, China, we aimed to assess long-term antibody response at 12 months after infection and comprehensively evaluate the structural and functional recovery of the lung and cardiovascular systems. We also attempted to identify potential risk factors associated with those long-term consequences.

## Results

Between January 15 through 31 March 2020, a total of 307 patients were diagnosed with COVID-19 at Xiangyang Central Hospital, which represented 55.9% of 549 cases in the downtown and 26.1% of 1175 cases city-wide. During hospitalization, 12 patients succumbed to COVID-19-induced respiratory distress or lethal infection, which translated to a mortality rate of 3.9% in line with the citywide mortality rate of 3.4% (40/1175). All 295 survivors were invited to participate in this study and the final cohort consisted of 121 survivors including 19 recovered from severe COVID-19 (Supplementary Fig. 1). Clinical procedures were performed at Xiangyang Central Hospital between 25 December 2020 and 29 January 2021.

### Demographic and clinical features of participants

Demographic-wise, this cohort consisted of middle-aged Chinese population with an overall comorbidity prevalence of 30.6%, including hypertension (25.6%) and diabetes (6.6%) as the most common preexisting conditions, which was typical for the local agricultural and industrial population with a preference of high-salt diets (Table 1). The participants of this study were among the earliest confirmed COVID-19 patients with virological confirmation dates as early as January 19, 2020. Standard of care consisted of antivirals, antibiotics, immunomodulants and supplemental oxygen was given to participants following CDC guidelines (Supplementary Table 1). Only 1 in this cohort received invasive ventilation (Supplementary Table 1), which reflected the dismal mortality rate among critically ill patients relying on respiratory support.^20^ Of note, the basic characteristics of this cohort were comparable with the entire population of COVID-19 survivors treated at this hospital (Supplementary Table 2).

**Table 1:** Characteristics of participants by COVID-19 severity

	No./total No. (%) or median (IQR)			*P*^a^
	Overall (*n* = 121)	Non-severe (*n* = 102)	Severe (*n* = 19)	
*Demographic characteristics*				
Age, years	49 (40–57)	48.5 (39–57)	56 (46–59)	0.109
<30	6 (5)	6 (5.9)	0 (0)	0.588
>60	14 (11.6)	11 (10.8)	3 (15.8)	0.460
Female	71 (58.7)	63 (61.8)	8 (42.1)	0.132
Body mass index	23.9 (22.5–25.6)	23.9 (22.6–25.4)	24.2 (21.9–26)	0.859
Comorbidity	37 (30.6)	29 (28.4)	8 (42.1)	0.280
Hypertension	31 (25.6)	26 (25.5)	5 (26.3)	1.00
Diabetes	8 (6.6)	6 (5.9)	2 (10.5)	0.610
Autoimmune diseases	2 (1.7)	1 (1)	1 (5.3)	0.290
Cardiovascular diseases	3 (2.5)	3 (2.9)	0 (0)	1.00
Cancer	1 (0.8)	1 (1)	0 (0)	1.00
*Last follow-up findings*				
Symptom onset to last follow-up, days	348 (344–351)	348 (344–351)	347 (339–351)	0.592
Discharge to last follow-up, days	316 (311–321)	317 (312–323)	312 (300–314)	<.001
Persistent COVID-19-related symptoms	36 (29.8)	21 (20.6)	15 (78.9)	<.001
Respiratory symptoms	22 (18.2)	10 (9.8)	12 (63.2)	<.001
Neurological/mental symptoms	15 (12.4)	9 (8.8)	6 (31.6)	0.014
Fatigue/weakness	14 (11.6)	7 (6.9)	7 (36.8)	0.001
Other disease diagnosis after discharge	12 (9.9)	9 (8.8)	3 (15.8)	0.400
SARS-CoV-2 PCR-positive after discharge	5 (4.1)	5 (4.9)	0 (0)	1.00
Anti-SARS-CoV-2 IgM positive	2 (1.7)	2 (2)	0 (0)	0.583
Weakly positive	3 (2.5)	2 (2)	1 (5.3)	
Negative	116 (95.9)	98 (96.1)	18 (94.7)	
Anti-SARS-CoV-2 IgG positive	55 (45.5)	42 (41.2)	13 (68.4)	0.062
Weakly positive	20 (16.5)	17 (16.7)	3 (15.8)	
Negative	46 (38)	43 (42.2)	3 (15.8)	
Abnormal CT findings at prior follow-ups	21/62 (33.9)	12/48 (25)	9/14 (64.3)	0.010
Abnormal CT findings at last follow-up	10 (8.3)	0 (0)	10 (52.6)	<.001

^a^*P* values were calculated by Fisher’s exact test (data with only 1 row) or Chi-square test (multi-row data) for categorical variables, and by Mann–Whitney *U* test for continuous variables between the two groups

After stratifying the cohort by severity graded according to the guideline,^21^ severe groups had higher ages, less females, and more comorbidities (Table 1). Severe group also presented more symptoms at admission, and received more aggressive immunomodulatory therapies, supplemental oxygen, and ICU care during hospitalization (Supplementary Table 1). Both severe and non-severe groups share similar lengths since symptom onset, while the severe group had shorter periods since recovery because of longer hospitalization (Table 1).

### Long-lasting SARS-CoV-2 antibody response 1-year after infection

First, blood samples were screened by colloidal gold-based immunochromatographic assays (GICA) separately detecting IgM and IgG against SARS-CoV-2.^22^ At a median of 11 months post- infection, only 4% (95% CI, 2–10%) participants returned positive IgM results, which included both positive and weakly positive results, while 62% (95% CI, 54–71%) were IgG positive (Table 1), comparing to 82.2% prevalence of IgM among pre-discharge samples from the same hospital.^23^ Severe group showed higher prevalence of IgG, while the prevalence of IgM was equally low in both groups (Table 1).

**Fig. 1:**
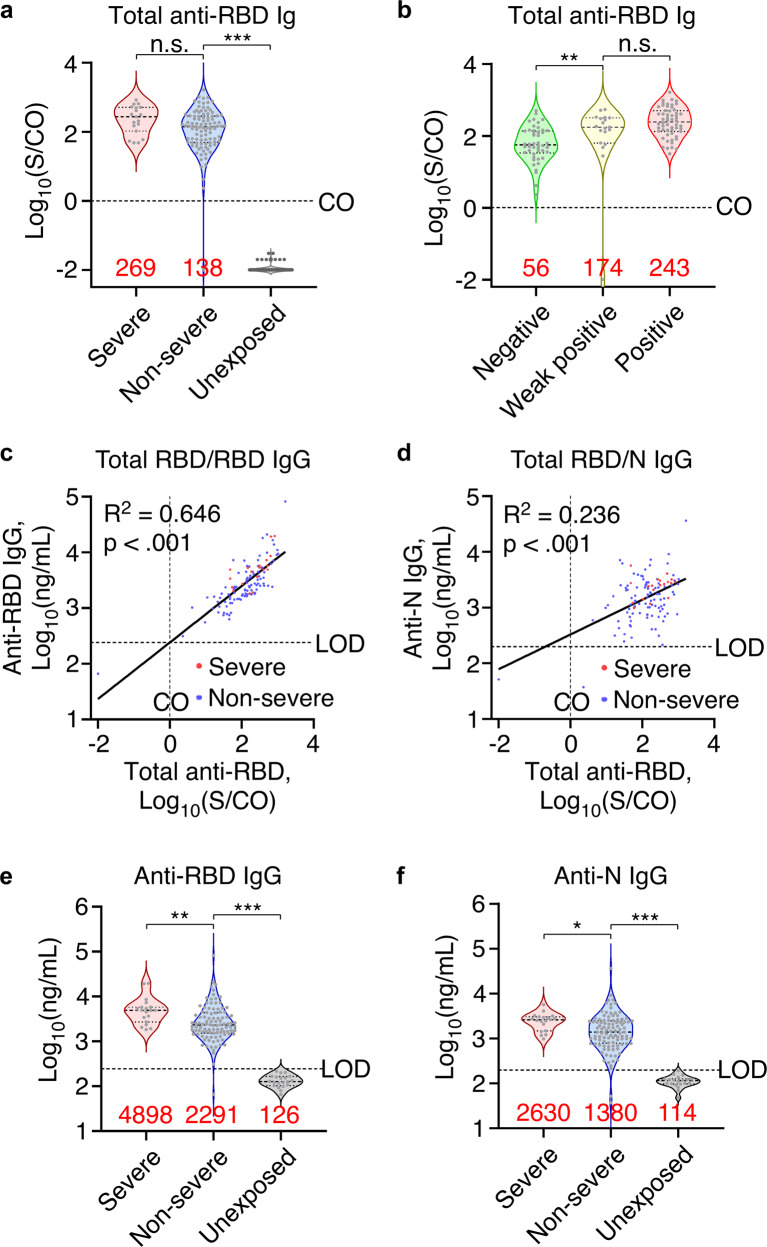
Long-lasting SARS-CoV-2 antibody response 1-year after infection. **a** Total anti-RBD antibodies in non-severe (*n* = 102) and severe patients (*n* = 19) sera 10–12 months after infection and in unexposed population (*n* = 100) measured by CMIA. CO, cutoff value. **b** Total anti-RBD antibodies in sera of participants with SARS-CoV-2 GICA negative (*n* = 46), weak positive (*n* = 19), or positive (*n* = 56) results. **c**, **d** Scatter plot showing linearity of total anti-RBD antibodies with anti-RBD IgG (**c**) or anti-N IgG (**d**). Linear regression was performed on all datapoints. *n* = 121. **e**, **f** Anti-RBD (**e**) and anti-N IgG (**f**) concentrations in non-severe (*n* = 102) and severe patients (*n* = 19) 10–12 months after infection and in unexposed population (*n* = 20) measured by ELISA. Red numbers indicate median value for violin plots. Dotted lines indicate the diagnostic cut-off (CO) for total anti-RBD antibody titers or the limit of detection (LOD) for anti-RBD or anti-N IgG concentrations. *p < .05, **p < .01, ***p < .001, n.s. not significant

Next, the concentration of total antibodies against the receptor-binding domain of SARS-CoV-2 spike protein (RBD) was quantitatively measured by chemiluminescence microparticle immunoassays (CMIA).^24^ Although signal/cutoff (S/CO) ratios were lower in non-severe group, all but 1 of the results were above the positive diagnostic threshold of S/CO = 1.0, when all 100 samples of unexposed individuals, which were randomly chosen from sera of in-hospital patients who had negative results from multiple PCR and serological tests for SARS-CoV-2 before and after the date of serum collection, had S/CO values <0.05 (Fig. 1a). Furthermore, five samples negative for both IgM and IgG in GICA showed S/CO values higher than the medium value of positive samples in CMIA, indicating higher sensitivity of CMIA than GICA (Fig. 1b).

Third, ELISA kits were used to detect anti-RBD IgG and anti-nucleocapsid protein (anti-N) IgG, which targeted the abundant component of SARS-CoV-2 with second high immunogenicity.^25^ The titer of total anti-RBD antibodies showed a log-linear relationship with anti-RBD IgG, and to a lesser degree with anti-N IgG regardless of disease severity (Fig. 1c, d). Similar with the CMIA results, severe group showed higher anti-RBD IgG and anti-N IgG concentrations than non-severe group (Fig. 1e, f). These quantitative data suggest a long-lasting antibody response up to 1-year after infection.

### Decay of antibody response in COVID-19 patients and general population

**Fig. 2:**
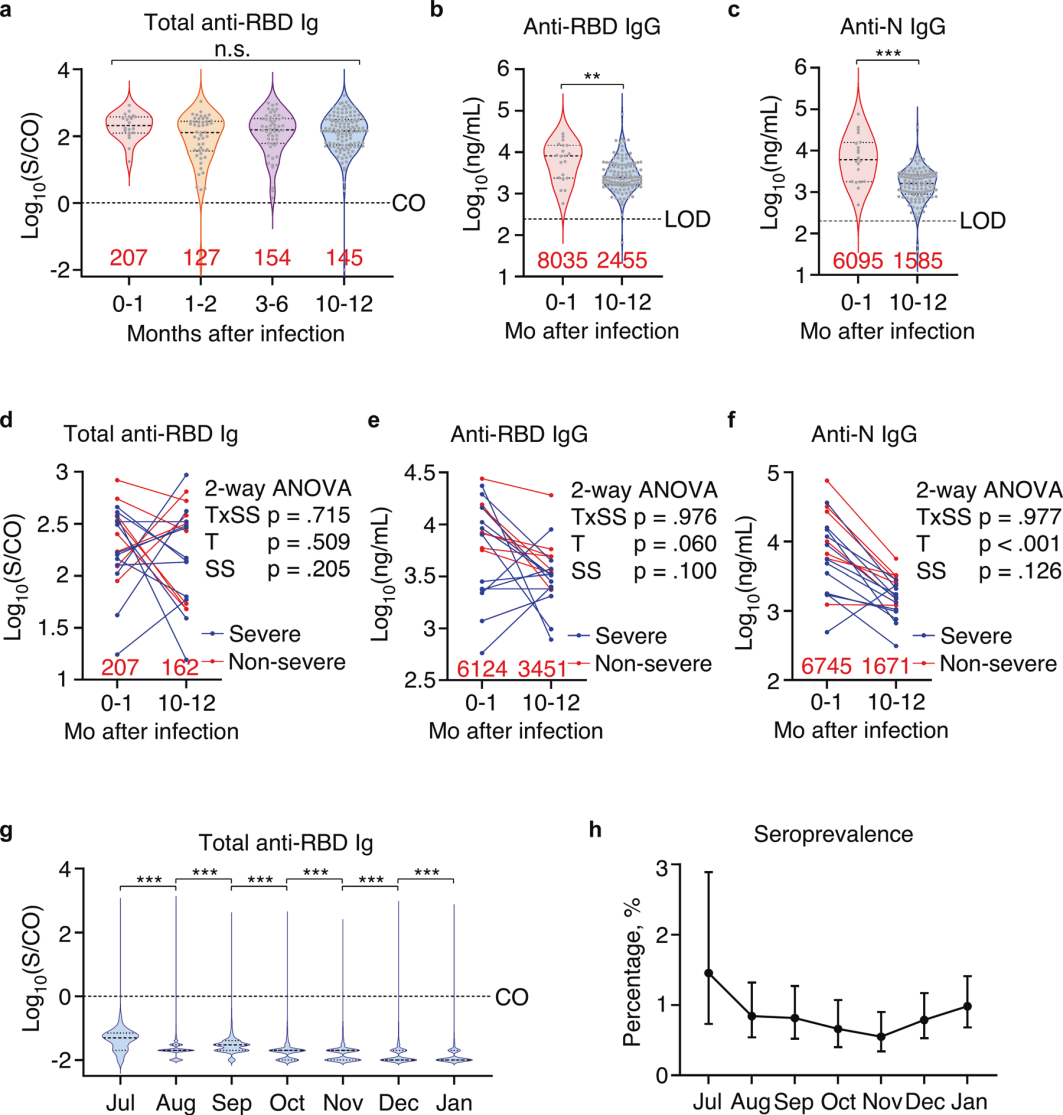
Decay of antibody response in COVID-19 patients and general population. **a** Total anti-RBD antibodies in 0–1-month post-infection (*n* = 20), 1–2-month post-infection (*n* = 50), 3–6-month post-infection (*n* = 54), and 10–12-month post-infection sera (*n* = 121) measured by CMIA. **b**, **c** Anti-RBD (**b**) and anti-N IgG (**c**) concentrations in 0–1-month post-infection (*n* = 20) and 10–12-month post-infection sera (*n* = 121). **d**–**f** Before-after plots of total anti-RBD antibodies (**d**), anti-RBD (**e**) and anti-N IgG (**f**) concentrations in 0–1-month post-infection and 10–12-month post-infection sera from the sample patients. Data were analyzed by matched two-way ANOVA and *p* values for time effect (T), disease severity scale (SS), and their interactions (TxSS) were reported on the side. *n* = 20. **g** Monthly results of total anti-RBD antibodies in patients without COVID-19 history and admitted to Xiangyang Central Hospital. *n* = 550 (Jul), 2258 (Aug), 2333 (Sep), 2426 (Oct), 2912 (Nov), 3052 (Dec) and 2953 (Jan 2021). **h** Monthly seroprevalence with 95% CI based on CMIA results in patients admitted to Xiangyang Central Hospital. Red numbers indicate median value for violin plots. Dotted lines indicate the diagnostic cut-off (CO) for total anti-RBD antibody titers or the limit of detection (LOD) for anti-RBD or anti-N IgG concentrations. **p < .01, ***p < .001, n.s. not significant

These participants have also been enrolled in prior clinical trials and their serum samples collected during the trials were re-analyzed in this study. Among them, 20, 50, and 54 had samples collected within the 1 month, 1–2 months, and 3–6 months after infection, respectively. Across these 4 time points in 1-year span, total anti-RBD antibodies showed no significant variation (Fig. 2a). However, ELISA results showed decrease of both anti-RBD and anti-N IgG titers in 10–12-month post-infection samples comparing to 0–1-month post-infection samples (Fig. 2b, c). Similarly, in before-after comparison of identifiable 0–1-month and 10–12-month post-infection samples, there were comparable up and downs in total anti-RBD levels while most showed decreasing concentrations for anti-RBD IgG and anti-N IgG (Fig. 2d–f). We reasoned that since these 0–1-month post-infection samples were collected at a median of 19.5 (IQR, 14.5–29.25) days after disease onset and 14 (IQR, 10.5–22) days after diagnosis and admission, their antibody response was in an evolving state when seroconversion for IgM and IgG occurred simultaneously or sequentially.^26^

We next sought to assess the antibody response in general population of Xiangyang city, after 10 months without newly diagnosed COVID-19 patients. Between July 2020 to January 2021, 16,484 inpatients admitted to Xiangyang Central Hospital without prior COVID-19 diagnosis, which were deemed to represent the general population of Xiangyang City, were screened for SARS-CoV-2 antibodies by CMIA. Monthly CMIA results showed a gradual decrease of median antibody levels (Fig. 2g). The overall seroprevalence was 0.8% (95% CI, 0.7–0.9%) and monthly seroprevalence was gradually decreasing from July to November with a slight rise afterward due to new sporadic COVID-19 outbreaks in winter (Fig. 2h). These results indicate that community immunity from random exposure was rapidly diminishing after initial outbreak, while asymptomatic and non-hospitalized COVID-19 still gave rise to long-lasting immunity.

### Time- and variant-dependent decline of neutralization capacity

**Fig. 3:**
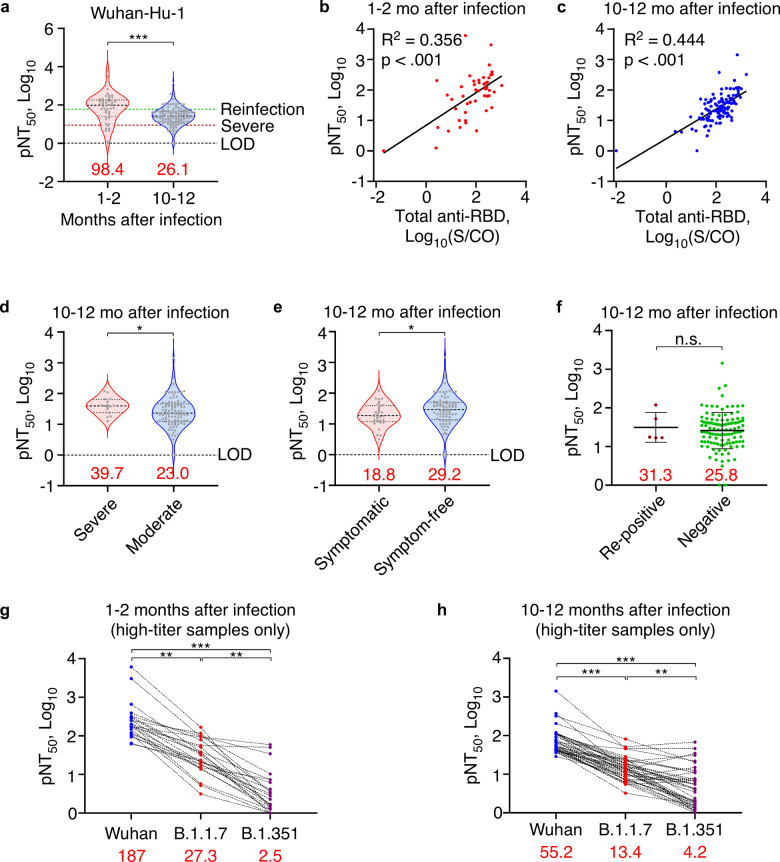
Time- and variant-dependent reduction of serum neutralization levels. **a** Neutralization levels of 1–2-month post-infection (*n* = 50) and 10–12-month post-infection sera (*n* = 119) against Wuhan-Hu-1 pseudovirus measured by PNA. Green and crimson dotted lines indicate 50% protective threshold against detectable and severe re-infection, respectively. **b**, **c** Scatter plot showing linearity of neutralization titers against Wuhan-Hu-1 pseudovirus with total anti-RBD antibodies in 1–2-month post-infection (*n* = 50) (**b**) or 10–12-month post-infection sera (*n* = 119) (**c**). Linear regression was performed on all datapoints after log-transformation. **d** Neutralization levels of 10–12-month post-infection sera against Wuhan-Hu-1 pseudovirus among severe (*n* = 18) and moderate COVID-19 survivors (*n* = 101). **e** Neutralization levels of 10–12-month post-infection sera against Wuhan-Hu-1 pseudovirus among symptomatic (*n* = 34) and symptom-free COVID-19 survivors (*n* = 85). **f** Neutralization levels of 10–12-month post-infection sera against Wuhan-Hu-1 pseudovirus among viral RNA re-positive (*n* = 5) and consistently negative COVID-19 survivors (*n* = 114). Bar = mean ± SD. **g**, **h** Before-after plots of neutralization levels against Wuhan-Hu-1, B.1.1.7 or B.1.351 pseudovirus in 1–2-month post-infection (*n* = 24) (**g**) or 10–12-month post-infection sera (*n* = 42) (**h**). Data were analyzed by Friedman test with post hoc comparisons. Red numbers indicate median value for violin plots or mean value for scatter plots. Dotted lines indicate the diagnostic cut-off (CO) for total anti-RBD antibody titers or the limit of detection (LOD) for neutralization assays. *p < .05, **p < .01, ***p < .001, n.s. not significant

To assess the protection levels at 1-year post-infection, we measured the neutralization capacity of serum samples collected at 1–2 months or 10–12 months after infection using a pseudovirus neutralization assay (PNA) based on the neutralization of WT (Wuhan-Hu-1) SARS-CoV-2 spike pseudotyped lentivirus-mediated transfection of a luciferase reporter in replication-deficient 293T-ACE2 cells.^27^ Two serum samples were not analyzed due to insufficient volume for PNA. Pseudovirus neutralization titers (pNT_50_) of 10–12-month post-infection samples were fourfold lower than pNT_50_ of 1–2-month post-infection samples (Fig. 3a), and pNT_50_ were positively correlated with total anti-RBD antibody levels in samples of both time points (Fig. 3b, c). A recent predictive model estimates the neutralization level for 50% protection to be 20.2% of the mean convalescent level against detectable SARS-CoV-2 infection and 3% of the mean convalescent level against severe infection.^28^ Applying pNT_50_ of 1–2-month post-infection samples as the convalescent neutralization level, we calculated that 17% (95% CI, 11–24%) and 87% (95% CI, 80–92%) participants were 50% protected against detectable and severe re-infection of WT SARS-CoV-2 at 1-year after first infection, respectively. Similar with total and IgG fraction of anti-SARS-CoV-2 antibodies, neutralization level was higher among participants with severe infections while lower among those with persistent symptoms (Fig. 3d, e). Interestingly, participants with viral RNA re-positive events after discharge showed normal neutralization and antibody levels at 1-year after initial infection (Fig. 3f, Supplementary Fig. 3a–c). Furthermore, we tested neutralization capacity against two WHO Variant of Concern, the B.1.1.7 strain (Alpha) first documented in UK and the B.1.351 strain (Beta) first documented in South Africa. Due to the detection limitation of PNA and known immune evasiveness of variant strains,^29^ we only tested 42 recovery phase serum samples and 24 convalescent-phase serum samples with highest neutralization titers against WT SARS-CoV-2. In both 1–2-month and 10–12-month post-infection samples, the pNT_50_ was significantly reduced comparing to the pNT_50_ for WT SARS-CoV-2, which was especially alarming to observe more than 10-fold decrease against the immune evasive B.1.351 strain (Fig. 3g, h). These data suggest that although neutralizing antibody still offer protection against severe infections in 87% COVID-19 survivors up to 1-year after infection, they were less effective against variants, especially those with strong immune evasiveness.

### Factors associated with long-term antibody response

Non-parametric correlation tests were conducted to screen for demographic and clinical factors correlated with the total anti-RBD antibody titer. At *α* = 0.05, age (*ρ* = 0.191, *p* = 0.036), severe disease (*ρ* = 0.202, *p* = 0.026), lymphopenia at admission (*ρ* = 0.194, *p* = 0.033), supplemental oxygen (*ρ* = 0.193, *p* = 0.034) and ICU care (*ρ* = 0.192, *p* = 0.035) were found to be independently associated with the total anti-RBD antibody titer, while the later 3 factors closely correlated with disease severity. Interestingly, age was positively associated with total and IgG fractions of anti-RBD antibodies and neutralization titers (Supplementary Fig. 2a–c). Model-based generalized linear regression suggested that total anti-RBD antibodies were positively associated with age, severe disease, time from discharge and LOS, and negatively associated with persistent symptoms and male sex (Table 2). In contrast, anti-RBD IgG titers were associated with the same group of factors except time from discharge and LOS, while anti-N IgG titers were only associated with age and severe disease, both positively (Table 2).

**Table 2:** Parameters of regression models

Factors and covariates	*β*	95% CI		*P*
		Lower	Upper	
*Total anti-RBD Ig as dependent variable*^a^				
Severe disease	0.670	0.279	1.060	0.001
Male sex	−0.238	−0.428	−0.047	0.015
Persistent symptoms	−0.351	−0.574	−0.129	0.002
Glucocorticoids	−0.217	−0.601	0.167	0.267
Interferons	0.258	−0.022	0.538	0.071
Age	0.012	0.004	0.020	0.003
Length of stay	0.030	0.011	0.049	0.002
Time from discharge	0.030	0.012	0.047	0.001
*Anti-RBD IgG as dependent variable*^*a*^				
Severe disease	0.442	0.150	0.735	0.003
Male sex	−0.151	−0.293	−0.008	0.038
Persistent symptoms	−0.211	−0.378	−0.045	0.013
Glucocorticoids	−0.079	−0.366	0.209	0.591
Interferons	0.047	−0.163	0.256	0.662
Age	0.010	0.004	0.016	0.001
Length of stay	0.012	−0.002	0.026	0.101
Time from discharge	0.010	−0.003	0.024	0.120
*Anti-N IgG as dependent variable*^*a*^				
Severe disease	0.399	0.093	0.705	0.011
Male sex	0.006	−0.143	0.156	0.936
Persistent symptoms	−0.164	−0.338	0.010	0.065
Glucocorticoids	−0.133	−0.433	0.168	0.388
Interferons	0.032	−0.188	0.251	0.778
Age	0.010	0.003	0.016	0.003
Length of stay	0.001	−0.014	0.016	0.925
Time from discharge	0.002	−0.011	0.016	0.725

^a^All antibody concentrations were log10-transformed

### Differential functional recovery from severe COVID-19 and risk factors

**Fig. 4:**
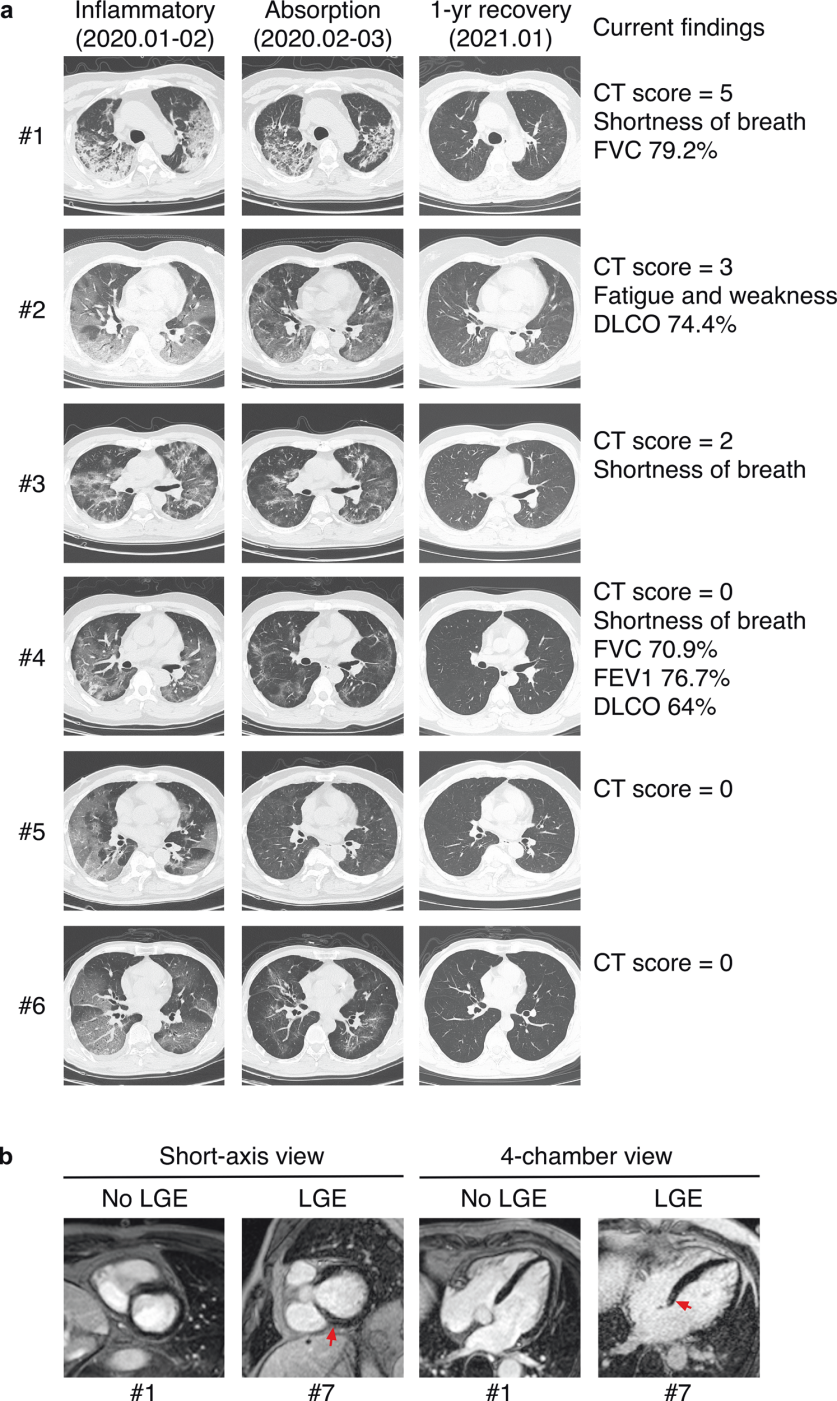
Chest CT and cMRI showed differential recovery of severe COVID-19 patients. **a** Representative chest CT images and related findings of participants who have recovered from severe COVID-19. Images in the left column show wide-spreading lung inflammation during the acute phase. Images in the middle column show partial absorption of lung lesions during early recovery. Images in the right column show structural recovery of the lung 10–12 months after infection. CT score of the right column images, symptomatic, and PFT findings were also listed on the right. **b** Representative cMRI images of focal myocardial fibrosis and images without findings. PSIR sequences in short-axis view and 4-chamber view showed focal LGE in the left ventricular midwall (red arrows)

At 1-year after infection, 30% participants and 79% of those with severe COVID-19 are still experiencing at least one of COVID-19-related symptoms (Table 1). Chest CT scans showed that 8% participants with current abnormalities, comparing to 34% prevalence among 62 patients who provided CT reports from previous follow-up checks between May and September 2020 (Table 1). No non-severe patients showed chest CT abnormalities, while 53% severe patients still showed various abnormalities with ground-glass opacities and interstitial fibrosis as the most frequent findings (Fig. 4a).

Six of the 11 participants underwent PFT showed abnormal findings indicating a restrictive pattern of lung change (Table 3). The most common abnormal value was reduced DLCO (<80% of predicted), which indicated interstitial lung disease that matched radiological findings except for one participant, who showed normal CT images but significant impairment of lung function (Fig. 4a). All participants with abnormal PFT findings reported persistent COVID-19-related symptoms; however, their antibody response was not lower than those with normal PFT results (log_10_[median], total anti-RBD 2.23 vs 2.31, *p* = 1.00; anti-RBD IgG 3.69 vs 3.65, *p* = .398). All participants reached normal range in six-minute walk test and 89% severe patients reported 0 in mMRC questionnaire (Table 3), suggesting a relatively low level of physical impairment.

**Table 3:** Functional recovery of severe patients

	No./total No. (%) or median (IQR)
*Pulmonary function test findings (n* *=* *11)*	
Abnormal findings	6 (54.4)
FVC, L	3.40 (3.06–4.03)
% of predicted	94.1 (83.5–99.0)
<80% of predicted	2 (18.2)
FEV_1_, L	2.91 (2.49–3.42)
% of predicted	94.3 (85.3–101)
<80% of predicted	1 (9.1)
FEV_1_/FVC, %	84.5 (82.1–85.7)
<70%	0 (0)
DLCO, mmol/kPa/min	7.08 (6.31–8.36)
% of predicted	81.8 (73.2–89.2)
<80% of predicted	4 (36.4)
DLCO/VA, mmol/kPa/min/L	1.50 (1.40–1.57)
% of predicted	95.4 (88.2–104)
<80% of predicted	1 (9.1)
Current abnormal CT findings	7 (63.6)
Current respiratory symptoms	9 (81.8)
*Six-minute walk test (n* *=* *13)*	
Distance, m	545 (518–625)
% of predicted	93.8 (79.3–97.9)
<lower limit of normal range	0 (0)
*mMRC dyspnea scale (n* *=* *19)*	
0	17 (89.5)
1	1 (5.3)
2	1 (5.3)
*Heart abnormalities*	
Abnormal electrocardiogram	3/19 (15.8)
Abnormal echocardiography	6/19 (31.6)
Reduced LV diastolic function	6/19 (31.6)
Valve disfunction	2/19 (10.5)
Abnormal cardiac MRI	4/6 (66.7)
LV fibrosis (LGE)	2/6 (33.3)
Left atrial enlargement	3/6 (50)
Right atrial enlargement	1/6 (16.7)
LV hypertrophy	1/6 (16.7)
Reduced LV ejection fraction	0/6 (0)
Taking antihypertensives	6/19 (31.6)
SBP = 130 mmHg or higher	4/19 (21.1)
DBP = 80 mmHg or higher	7/19 (36.8)
*Blood test findings (n* *=* *19)*	
Complete blood count findings	2 (10.5)
RBC < 4.3 × 10^9^/L	2 (10.5)
Hemoglobin < 30 g/L	2 (10.5)
Inflammation-related findings	2 (10.5)
hs-CRP > 0.5 mg/L	0 (0)
Transferrin < 2.3 g/L	2 (10.5)
BNP > 100 pg/mL	1 (5.3)
D-dimer > 0.55 mg/L	0 (0)
Liver-related findings	2 (10.5)
High alanine transaminase^a^	2 (10.5)
High aspartate transaminase^a^	1 (5.3)
Kidney-related findings	4 (21.1)
High creatinine^a^	3 (15.8)
High urea^a^	2 (10.5)

*FVC* forced vital capacity, *FEV1* forced expiratory volume in 1 s, *DLCO* diffusing capacity of the lungs for carbon monoxide, *VA* alveolar volume, *mMRC* modified Medical Research Council, *LV* left ventricular, *LGE* late gadolinium enhancement, *SBP* systolic blood pressure, *DBP* diastolic blood pressure, *RBC* red blood cell, *hs-CRP* high sensitivity C-reactive protein test, *BNP* brain natriuretic peptide

^a^Results exceeded higher limits of normal ranges

Among the six participants with reduced left ventricular (LV) diastolic function, two showed late gadolinium enhancement (LGE) in LV walls (Fig. 4b), which indicated scar formation and myocardial fibrosis.^30,31^ However, all six participants showed normal LV ejection fraction, suggesting that ventricular remodeling was still in the early stage. Of note, 5 participants of cMRI were also receiving medical treatment of hypertension, and thus it is difficult to rule out the contribution of hypertension in the development of cardiac fibrosis.

Laboratory tests showed that inflammation markers were lower in all severe participants (Table 3). Two participants were also diagnosed with amenia, liver dysfunction and kidney failure. Other participants with severe COVID-19 did not show more than 1 related abnormalities in these laboratory tests. These imaging, functional and laboratory examinations in together indicated low incidence of long-term consequences in non-severe COVID-19 but also suggested a differential recovery pattern in severe cases, especially those with preexisting conditions.

## Discussion

Long-time recovery and rehabilitation of COVID-19 is a contentious topic partly because of the sheer number of patients over the world with drastically different levels of medical resources.^32,33^ We believe that studying early Chinese patients offers a unique insight into this matter because they were proactively treated in hospitals thank to the large capacity of municipal hospitals.^34^ Timely in-hospital treatment not only gives better care,^35^ but also leaves unbiased documentation of the full disease course, which improves the quality of data and future analyses. In this study, we examined immunity and recovery status in 121 patients, which was about half of survived COVID-19 patients in the Central Hospital. Long-lasting immunity against SARS-CoV-2 in the form of anti-RBD IgG were found in 99% recovered patients, and close to 90% of them were 50% protected against severe re-infection at 1-year after the first infection. Comparing to convalescent samples, total anti-RBD antibodies were stable at 1-year after infection, while anti-RBD IgG and neutralization levels showed a threefold decrease. The longevity of antibody response was associated with known factors such as disease severity and sex, while we found a positive correlation between age and antibody response, and persistent symptoms as an indication of weaker immune response. Non-severe patients in general have recovered well from COVID-19 with no chest CT abnormalities and less symptoms. However, recovery statuses of severe patients varied significantly with cases of complete recovery without symptoms and also those with multi-organ complications that impaired daily life. Although weak immune response was associated with persistent symptoms, it was not associated with functional recovery of the lung. In addition, cMRI identified two cases of left ventricular wall fibrosis without reduced ejection fraction among six severe patients with cardiac abnormalities in ECG or Echo.

An interesting finding of this study is the positive correlation between age and antibody levels, which is against the common belief that elders have a less effective immune system. We ruled out the possibility that older patients were prone to severe disease which led to stronger immune response by finding similar correlations after excluding severe cases. Screening of all factors correlated with age revealed that older patients tended to be infected later than younger ones. Since most earlier cases in Xiangyang were young to mid-aged travelers from Wuhan, who had been visiting parents or senior relatives for the upcoming Lunar New Year, it is possible that these older patients living with asymptomatic or mild patients had longer exposure to virus than the younger ones who might got infected earlier by incidental contacts with a spreader. It is also possible that mutation of spike protein increased its immunogenicity in the second wave of patients.^36^ Furthermore, more elders were taking anti-hypertensives including angiotensin-converting enzyme inhibitors (ACEi), which may have affected viral load and immunogenicity.^37^ Nevertheless, this result implies that elder population may still get the full benefit of vaccination.

Another intriguing point of the data is the lack of association between antibody response and reoccurrence of positive SARS-CoV-2 viral test after discharge. Weak immune response and subsequent incomplete viral clearance were proposed as the cause of such “re-positive” event.^38^ However, large-scale studies found no significant difference of antibody titers in these re-positive patients.^8,9^ We observed five cases with re-positive history, including one patient with four re-positive events after discharge. In all five cases, their mean anti-RBD antibodies and neutralization titers were comparable with other participants without re-positive events. Shared attributes of these cases were female, non-severe disease, age between 30 and 50 years old, diagnosed before 15 February 2020, and lived in or visited Wuhan recently. All of them recovered well and reported no COVID-19-related symptoms. Since the infectivity of re-positive individuals is not clear,^9^ further study into the virology of SARS-CoV-2 is needed to uncover the underlying mechanism and reduce the risk of asymptomatic transmission.

Our data showed that persistent COVID-19-related symptoms were associated with low neutralization titers and antibody concentrations, while the lack of virological traces even during post-discharge symptom onset and the fact that relapse of COVID-19 after full recovery and without any epidemiologic contact has not yet been reported argue against SARS-CoV-2 latency as the cause of these symptoms. We instead speculate that the immune system of these individuals is less efficient in response to viral infection at the respiratory tract, which makes them prone to respiratory tract infections of other microbes that cause symptoms similar with COVID-19.

Long-lasting COVID-19-related symptoms after virological clearance, also known as “long COVID-19”, are a worrisome feature of post-COVID-19 rehabilitation,^39,40^ while their causal relationship with COVID-19 or SARS-CoV-2 infection is debatable due to the contribution of preexisting conditions. In this study, we define COVID-19-related symptoms as those only appeared after or shortly before the onset of COVID-19 and found about 30% participants still experiencing at least one of them at 1-year post-infection, which is in line with large surveys from Germany (34.8% prevalence at 7-month, *n* = 958) and Italy (40.2% at 6-month, *n* = 599).^41,42^ Our finding of 79% participants with severe COVID-19 still reporting symptoms at 1-year post-infection is also reminiscent of the follow-up study of patients treated at Jin Yin-tan Hospital in Wuhan City (76% at 6-month, *n* = 1,733).^11^ It is known that COVID-19 could have neurological and psychiatric manifestation,^43,44^ and we suspect that some symptoms not associated with any serological indications or functional recovery status could be the result of stress or even PTSD-like psychiatric disorders as suggested in a survey of COVID-19 patients in the UK.^45^ While these psychiatric disorders might not be caused by SARS-CoV-2 directly, they are likely more common than we expected due to their general lack of clinical appreciation, and further study of the clinical efficacy of mental health intervention might help addressing this global healthcare issue.

This study has several limitations. First, the baseline data of most functional tests were not available due to limited testing functionality or prohibition such as PFT during the initial outbreak, which made it difficult to rule out confounders such as preexisting conditions. However, chest CT imaging of all participants in acute and recovery phases were retrieved for comparison. Second, a number of functional test results were confounded by underlying conditions and cannot be causally attributed to COVID-19. Namely, the cardiac findings in this cohort may be caused by hypertension instead of COVID-19. Third, eight severe patients declined to take PFT or six-minute walk test for personal reasons such as fear of infection via spirometer, which may lead to bias or missing information. Forth, longitudinal data regarding the antibody response or functional recovery are lacking or limited to two-time points. Last, regression analyses were partially based on retrospective data which limited the interpretation of results.

In summary, our study provides clinical evidence of a long-lasting antibody response in recovered COVID-19 patients while highlights the immune evasiveness of SARS-CoV-2 variants, which urges caution among previously infected and immunized population. The differential recovery pattern also warrants further research into the pathophysiology and rehabilitation of COVID-19.

## Materials and methods

### Study design and participants

This prospective observational cohort study enrolled recovered COVID-19 patients diagnosed at Xiangyang Central Hospital, located in Xiangyang city of Hubei province, during January 15 through 31 March 2020. COVID-19 diagnosis was confirmed by two consecutive positive results of quantitative PCR-based SARS-CoV-2 nucleic acids tests (Sansure Biotech) of throat or nasal swab samples taken at two-time points separated by at least 24 h. Severe COVID-19 was defined by the National Health Commission of China (NHC) Guideline for COVID-19 Diagnosis and Treatment as those with any of the following indications: respiration rate equal to or higher than 30 per minutes, resting oxygen saturation equal to or less than 93%, partial pressure of oxygen or fraction of inspired oxygen equal to or less than 300 mmHg, chest CT showing more than 50% increase of lesion area within 48 h, respiratory distress, shock, or ICU admission.^21^ All COVID-19 patients discharged from hospital met all the following criteria according to the NHC guideline: (a) two negative quantitative PCR-based SARS-CoV-2 nucleic acids tests of throat or nasal swab samples taken at two-time points separated by at least 24 h, (b) significant resolve or absorption of lung lesions in chest CT scans, (c) significant improvement of respiratory symptoms, and (d) no fever for 3 consecutive days.^21^ All patients survived COVID-19 and with a current phone number associated with their inpatient records were contacted by physicians via phone calls in December 2020, and those agreed to participate were scheduled for hospital visits at Xiangyang Central Hospital in December 2020 or January 2021.

The following patients were excluded from the study: (a) those who died before the study began, (b) those currently admitted in other hospitals, (c) those with mental, cognitive and other conditions that precluded informed consent, (d) those not living in or traveling outside of Xiangyang and not available during the study period, (e) those unable to be contacted, and (f) those declined to participate due to other personal reasons.

The human research protocol used by this study was approved by the Medical Ethics Committee of Xiangyang Central Hospital (certificate #: 2021–003) prior to data collection. All patient identifications were replaced by anonymous codes during record abstraction and analyses as stipulated by the Declaration of Helsinki. Informed consent forms were signed by all study participants.

### Clinical procedures

All participants received SARS-CoV-2 antibody tests based on colloidal gold-based immunochromatographic assays (Livzon Diagnostics), chemiluminescence microparticle immunoassay (InnoDx) and ELISA (Proteintech) and chest CT scans (Philips Ingenuity). Participants with severe COVID-19 history were assigned in-depth laboratory and functional tests for the lung and the heart. All procedures were performed by trained professionals following standard operating procedure or manufacturer’s instructions. Participants who had prior follow-up checks at Xiangyang Central Hospital were asked for consent before retrieving their frozen serum samples collected in March 2020 for antibody analyses. Serological data of recovered patients from the same cohort who have participated in another trial were also retrieved for analysis without personal identification.

On the day of scheduled visit, participants were first briefed on the study design and their individual agenda before being given the informed consent form. Those signed the consent were given standard physical examination, had blood drawing, and received chest CT scans. CT images were immediately analyzed by at least one radiologist and one pulmonologist for COVID-19-related abnormalities and calculated semi-quantitative scores of pulmonary involvements.^46^ Participants with non-severe COVID-19 history and no COVID-19-related CT findings were given optional rehabilitation and/or mental health counseling before being discharged.

Patients with severe COVID-19 history and those showing abnormal CT findings related to COVID-19 further received in-depth laboratory tests including complete blood count (Mindray), inflammation, metabolic, heart, liver, and kidney function combinations (Roche Diagnostics), electrocardiogram (Philips Pagewriter) and echocardiograph examinations (Philips EPIQ), and were invited to participate in lung function tests (Vyaire Medical MasterScreen PFT) and six-minute walk test^47^ before filling the mMRC dyspnea scale questionnaire.^48^ The lung function test was performed according to American Thoracic Society guidelines^49^ and made optional due to concerns regarding the contamination risk associated with the test.^50^ Patients with disabilities or advanced ages were given the option to opt out the six-minute walk test. Patients with abnormalities in echocardiographs were given cardiac MRI scans (Siemens MAGNETOM Aera), which would be performed on the second visit if it cannot be done during the first visit. All patients with abnormal findings were given medical consultation by physicians in relevant specialties and scheduled for further examination if necessary, before being discharged.

The COVID-19 history of all participants, including outpatient visits, pre-admission, inpatient, and follow-up records, were obtained from the Division of Medical Records of Xiangyang Central Hospital. Partially missing records of those patients who were later treated at other hospitals were requested and retrieved from Xiangyang Hospital for Infectious Diseases, Xiangyang Dongfeng Hospital, Xiangzhou District People’s Hospital, Xiangyang Hospital of Traditional Chinese Medicine, and Xiangyang First People’s Hospital. Records were abstracted in January 2021 by a trained team of two epidemiologists and five physicians into a standardized digital form based on the US CDC COVID-19 abstraction form with modifications to adapt local data and underwent daily quality control checks. Patient information was collected on COVID-19 diagnosis, patient demographics, comorbidities, prior high-risk exposure such as close contact to COVID-19 patients or visiting high-risk locations, initial vital signs and laboratory test results within 24 h of admission, CT images and reports, and temporary and long-term prescriptions to describe the cohort and as potential confounders.

### Pseudovirus neutralization assays

Neutralization of HIV pseudovirus carrying a luciferase reporter and encapsulated in WT (Wuhan-Hu-1), B.1.1.7, or B.1.351 spike proteins (GenScript) was measured by the reduction in *luc* gene expression in replication-incompetent HEK293T-ACE2 cells (GenScript). The 50% pseudovirus neutralization titer (pNT_50_) was defined as the serum dilution at which the relative light units (RLUs) were reduced by 50% compared with the top plateau value at lowest titrations or with control wells (virus + cells) when the top plateau was not reached after subtraction of the background RLUs of cell-only wells. The assay was performed according to the manufacturer’s protocol. In brief, pseudovirus was incubated with eight serial dilutions of each serum sample (fivefold for convalescent samples against WT pseudovirus, threefold for all other assays) for 1 h at room temperature. The mixture was then added to the culture of HEK293T-ACE2 cells in a 96-well plate and incubated in a humidified cell culture chamber at 37 °C with 5% CO_2_ for 48 h. The medium was removed at the end of incubation, and a one-step luciferase detection reagent (Fire-Lumi, GenScript) was prepared according to the manufacturer’s instruction and added to each well. Luminescence was measured by a luminometer (Fluoroskan FL, Thermo Scientific) after 3 min of incubation at room temperature. Samples without maximum RLU equal to 100 times of cell-only control were tested again with dilution of the initial sample when necessary. The pNT_50_ was calculated as EC50 with three or four parameters sigmoid function, depending on a comparison of fits, with bottom constrained to 0 and slope >0.

### Exposures

All participants were exposed to SARS-CoV-2 and diagnosed with COVID-19 during January to March 2020. During their COVID-19 disease courses, they have received combinations of therapies including antivirals, immunomodulatory agents, antibiotics, supplemental oxygen, and ICU care.

### Outcomes

The outcomes of this study were immunity against SARS-CoV-2 and functional recovery of the lung and other involved organs. Immunity against SARS-CoV-2 was assessed by multiple antibody assays. The colloidal gold-based test kit gave positive, weak positive, and negative readout of anti-SARS-CoV-2 IgM and IgG separately. The quantitative chemiluminescence microparticle immunoassay for antibodies against SARS-CoV-2 RBD was performed according to manufacturer’s protocol and previous publication,^24^ and the results were deemed positive if the signal/cutoff (S/CO) ratio ≥1. For ELISA tests, results were recorded and analyzed as continuous variables and the limit of sensitivity was calculated as mean + 2 × SD of 20 serum samples negative for SARS-CoV-2 antibodies in chemiluminescence assays. Functional recovery of the lung was assessed based on (1) current CT images comparing to images taken before discharge and during earlier follow-ups, (2) pulmonary function test results, and (3) six-minute walk test results. Recovery of the heart was assessed based on ECG, echocardiogram, and cardiac MRI scans. Recovery of other potentially involved organs were assessed by laboratory tests (Roche Diagnostics).

### Sample size

An initial target sample size of 108 was determined based on the assumption of a 1:5 ratio of severe and non-severe COVID-19 patient enrollment and *α* = 0.05. This sample size was calculated to have 90% power to detect a 10% difference of antibody concentrations. The final sample size exceeded the target in both groups.

### Statistical analysis

Quantitative data were presented in violin plots with all data points shown. Patient characteristics and clinical data were summarized as incidence with prevalence or median with IQR and were assessed with Fisher’s exact test (dichotomous variables) or *χ*^2^ test (variables with more than two categories) for categorical variables and Mann–Whitney *U* test for continuous variables. Antibody concentrations were log-transformed before being analyzed as continuous variables. The difference of antibody concentrations between groups were assessed by the Mann–Whitney *U* test (two groups) or Kruskal–Wallis test with post hoc comparisons (more than two groups). Special tests were mentioned in figure legends. Correlation was assessed by Spearman’s *ρ* test. Linearity between two factors was assessed by simple linear regression. Generalized linear models were used to assess factors associated with antibody titers. Analyses were performed using SPSS 26 (IBM) or Prism 9 (GraphPad). Missing data were excluded pairwise from analyses. Significance was evaluated at α = .05 and all tests were 2-sided. *p < 0.05; **p < 0.01; ***p < 0.001.

## Supplementary information

Supplementary Materials

## Data Availability

Reasonable requests for original dataset and clinical documents would be fulfilled by Dr. Peng Hong (peng.hong@downstate.edu).
