# Supplementary material for: SARS-CoV-2 immunity and functional recovery of COVID-19 patients 1-year after infection
Source: Signal Transduct Target Ther. 2021 Oct 13;6:368. doi: 10.1038/s41392-021-00777-z (PMC8512652; doi:10.1038/s41392-021-00777-z)
Supplement: Supplementary file 1 — Supplementary Materials [file 41392_2021_777_MOESM1_ESM.docx]

Supplementary Materials for

SARS-CoV-2 Immunity and Functional Recovery of COVID-19 Patients 1-Year After Infection

Yan Zhan, Yufang Zhu, Shanshan Wang, Shijun Jia, Yunling Gao, Yingying Lu, Caili Zhou, Ran Liang, Dingwen Sun, Xiaobo Wang, Zhibing Hou, Qiaoqiao Hu, Peng Du, Hao Yu, Chang Liu, Miao Cui, Gangling Tong, Zhihua Zheng, Yunsheng Xu, Linyu Zhu, Jin Cheng, Feng Wu, Yulan Zheng, Peijun Liu, Peng Hong

Correspondence to: Drs. Feng Wu, wufeng@hbuas.edu.cn,

Yulan Zheng, zhengyulan@hbuas.edu.cn,

Peijun Liu, liupeijun@hbuas.edu.cn, or

Peng Hong, peng.hong@downstate.edu

**This PDF file includes:**

Supplementary Tables 1 and 2

Supplementary Figures 1 to 3

| **Supplementary Table 1. COVID-19 clinical features and in-hospital care of participants** | | | | | |  |
| --- | --- | --- | --- | --- | --- | --- |
|  | | | No./total No. (%) or median (IQR) | | | *P*^*^ |
|  |  |  | Overall (n=121) | Non-severe (n=102) | Severe (n=19) |  |
| **Clinical features at admission** | | |  |  |  |  |
|  | Prior high-risk exposure | | 64 (52.9) | 50 (49) | 14 (73.7) | .078 |
|  | Symptom onset to hospitalization, days | | 5 (3-7) | 5 (3-7) | 7 (4-9) | .271 |
|  | Typical COVID-19 CT findings | | 116 (95.9) | 97 (95.1) | 19 (100) | 1.00 |
|  | Fever | | 108 (89.3) | 90 (88.2) | 18 (94.7) | .690 |
|  | Respiratory symptoms | | 72 (59.5) | 58 (56.9) | 14 (73.7) | .209 |
|  | Lymphopenia (<1.1×10^9^/L) | | 35 (28.9) | 21 (20.6) | 14 (73.7) | <.001 |
|  | Eosinopenia (<0.02×10^9^/L) | | 58 (47.9) | 45 (44.1) | 13 (68.4) | .078 |
|  | LDH >250 U/L | | 28 (23.1) | 15 (14.7) | 13 (68.4) | <.001 |
|  | CK >310 U/L | | 19 (15.7) | 13 (12.7) | 6 (31.6) | .078 |
|  | CK-MB >25 U/L | | 1 (0.8) | 1 (1) | 0 (0) | 1.00 |
| **In-hospital care** | | |  |  |  |  |
|  | Length of hospital stay (LOS), days | | 24 (19-30) | 23.5 (18-27) | 30 (25-40) | <.001 |
|  | Total disease duration, days | | 30 (24-37) | 29 (24-36) | 40 (32-55) | <.001 |
|  | Antiviral agents | | 108 (89.3) | 90 (88.2) | 19 (100) | .210 |
|  |  | Lopinavir/Ritonavir | 79 (65.3) | 61 (59.8) | 19 (100) | <.001 |
|  |  | Umifenovir | 43 (35.5) | 34 (33.3) | 9 (47.4) | .298 |
|  |  | Oseltamivir | 44 (36.4) | 36 (35.3) | 8 (42.1) | .609 |
|  |  | Ribavirin | 12 (9.9) | 9 (8.8) | 3 (15.8) | .400 |
|  | Immunomodulatory agents | | 36 (29.8) | 21 (20.6) | 15 (78.9) | <.001 |
|  |  | Glucocorticoids | 17 (14) | 3 (2.9) | 14 (73.7) | <.001 |
|  |  | Interferons | 18 (14.9) | 9 (8.8) | 9 (47.4) | <.001 |
|  |  | Chloroquine/Hydroxychloroquine | 14 (11.6) | 10 (9.8) | 4 (21.1) | .232 |
|  |  | Human Immunoglobulin | 11 (9.1) | 2 (2) | 9 (47.4) | <.001 |
|  | Antibiotics | | 85 (70.2) | 67 (65.7) | 19 (100) | .002 |
|  | Supplemental oxygen | | 15 (12.4) | 1 (1) | 14 (73.7) | <.001 |
|  |  | Invasive ventilation | 1 (0.8) | 0 (0) | 1 (5.3) | .157 |
|  | ICU admission | | 10 (8.3) | 0 (0) | 10 (52.6) | <.001 |
|  | Primarily treated at Central Hospital | | 59 (48.8) | 42 (41.2) | 17 (89.5) | .009 |
|  |  | Hospital for Infectious Diseases | 26 (21.5) | 26 (25.5) | 0 (0) |  |
|  |  | Dongfeng Hospital | 16 (13.2) | 15 (14.7) | 1 (5.3) |  |
|  |  | Xiangzhou District People's Hospital | 15 (12.4) | 14 (13.7) | 1 (5.3) |  |
|  |  | Hospital of Traditional Chinese Medicine | 3 (2.5) | 3 (2.9) | 0 (0) |  |
|  |  | First People's Hospital | 2 (1.7) | 2 (2) | 0 (0) |  |
| Abbreviations: IQR, inter quartile range; LDH, lactate dehydrogenase; CK, creatine kinase. | | | | | |  |
| **P* values were calculated by Fisher's exact test (data with only 1 row) or Chi-square test (multi-row data)  for categorical variables, and by Mann-Whitney U test for continuous variables between the 2 groups. | | | | | | |

| **Supplementary Table 2. Demographic and clinical characteristics of participants and all survivors** | | | | | |
| --- | --- | --- | --- | --- | --- |
|  | | | No./total No. (%) or median (IQR) | | *P*^*^ |
|  |  |  | Current cohort (n=121) | All survivors (n=295) |  |
|  | Age, years | | 49 (40-57) | 49 (38-58) | 0.793 |
|  | Female | | 71 (58.7) | 161 (54.6) | 0.514 |
|  | Comorbidity | | 37 (30.6) | 92 (31.2) | 1.000 |
|  |  | Hypertension | 31 (25.6) | 73 (24.7) | 0.901 |
|  |  | Diabetes | 8 (6.6) | 26 (8.8) | 0.557 |
|  | Severe COVID-19 | | 19 (15.7) | 54 (18.3) | 0.573 |
|  | Length of hospital stay, days | | 24 (19-30) | 23 (17-29) | 0.151 |
|  | Total disease duration, days | | 30 (24-37) | 29 (23-37) | 0.270 |
| **P* values were calculated by Fisher's exact test for categorical variables, and by Mann-Whitney U test for continuous variables between the 2 groups. | | | | | |

Supplementary Fig. 1


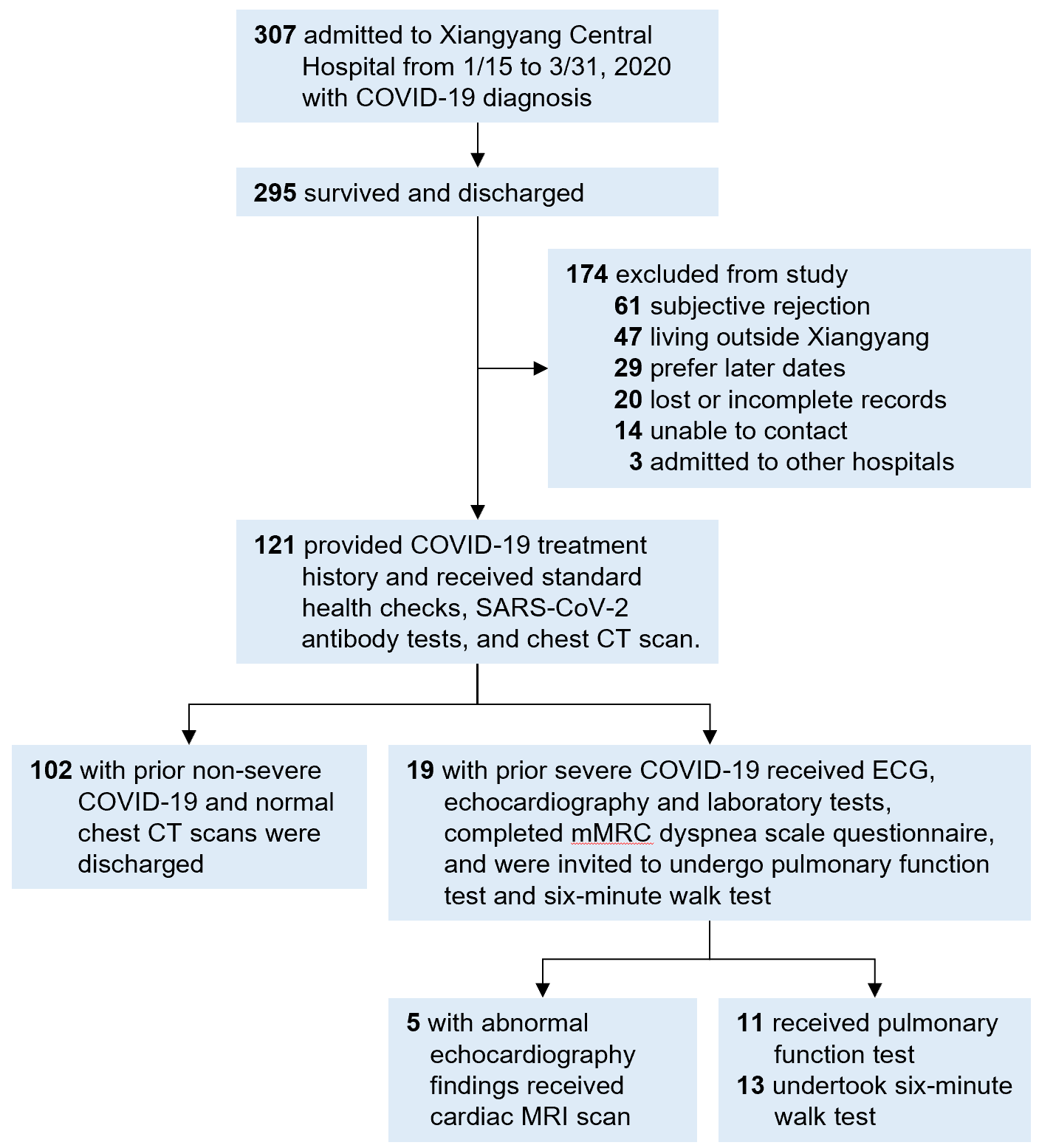


**Flow chart of COVID-19 patients admitted to Xiangyang Central Hospital.**

Supplementary Fig. 2


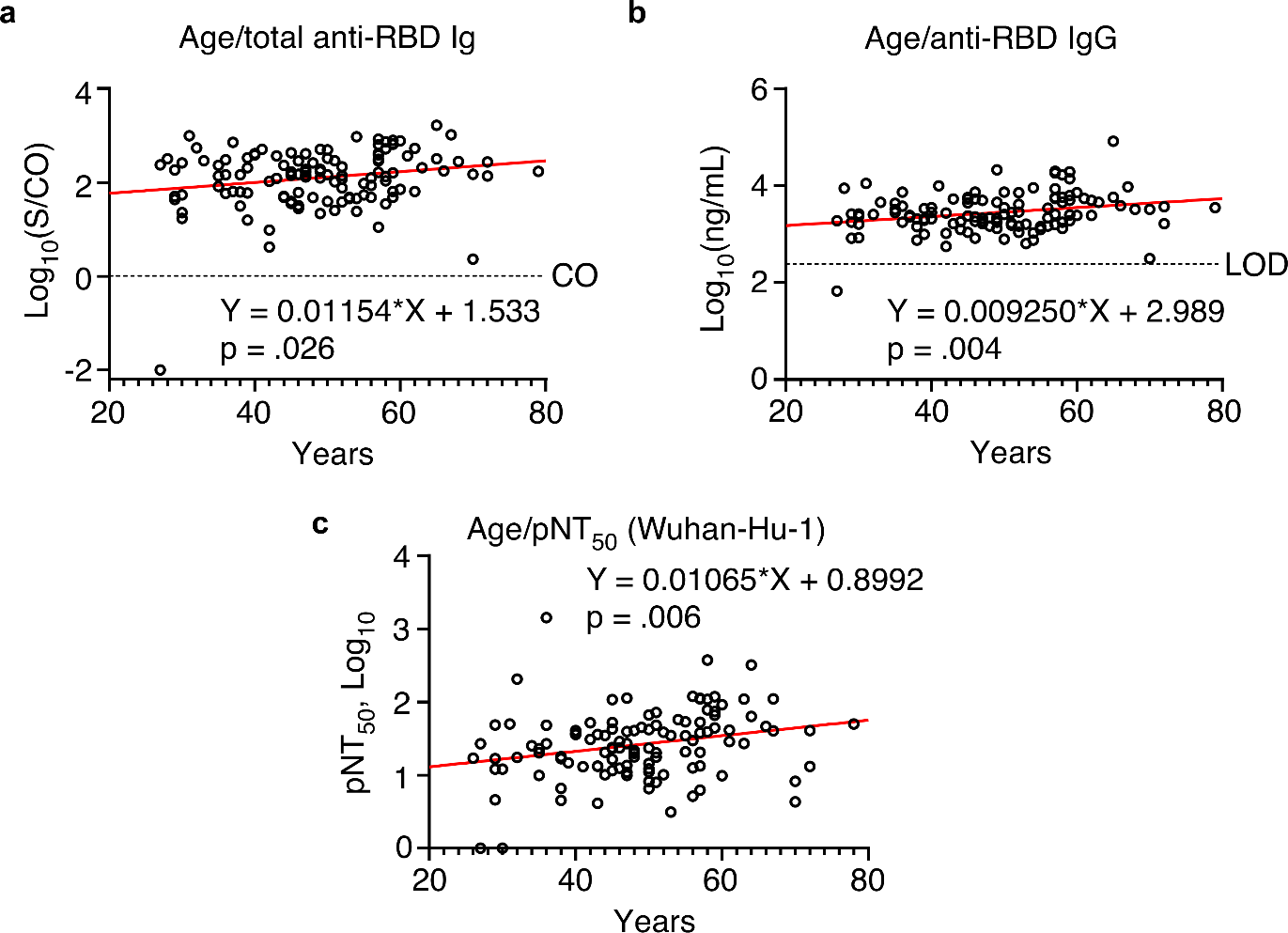


**Positive correlation of age with anti-RBD antibodies and neutralization titers.**

(**a-c**) Scatter plot showing linearity of age with total anti-RBD antibodies (a), anti-RBD IgG (b), or neutralization titers against WT pseudovirus (c). Dotted lines indicate diagnostic cut-off (CO) (a) or limit of detection (LOD) (b). n = 121.

Supplementary Fig. 3

**
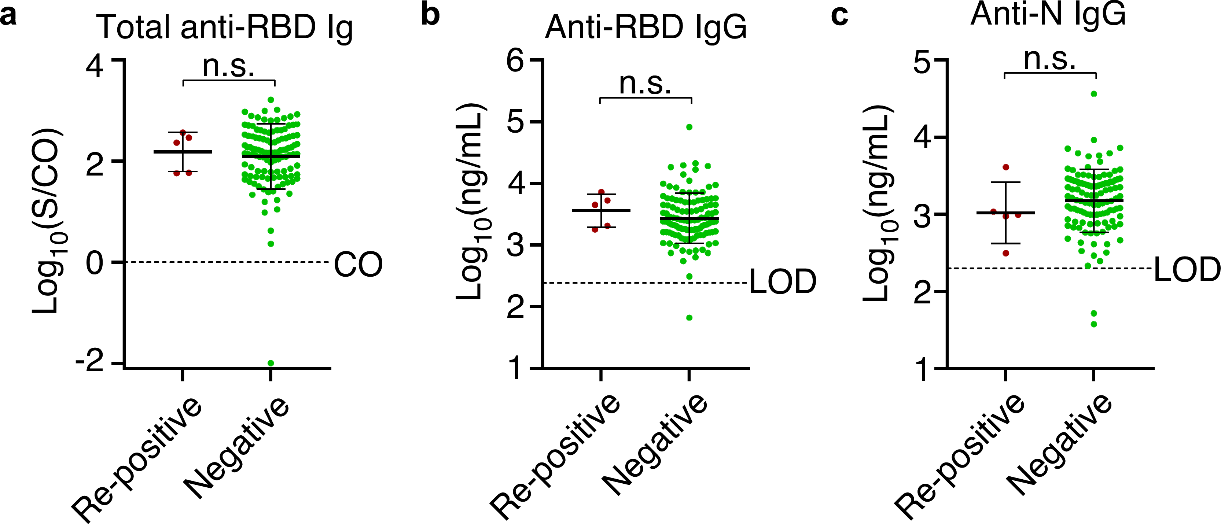
**

**Antibody response was not associated with viral RNA re-positive events.**

(**a-c**) Total anti-RBD antibodies (a), anti-RBD (b) and anti-N IgG (c) concentrations in patients with viral RNA re-positive events after discharge and in those never tested positive after discharge. Bar = mean ± SD. n = 5 (re-positive) and 116 (never).
